# Pleasantness makes a good time: musical consonance shapes interpersonal synchronization in dyadic joint action

**DOI:** 10.3389/fnhum.2024.1472632

**Published:** 2024-10-22

**Authors:** Giorgio Lazzari, Lucia Maria Sacheli, Charles-Etienne Benoit, Carlotta Lega, Floris T. van Vugt

**Affiliations:** ^1^Department of Brain and Behavioral Sciences, University of Pavia, Pavia, Italy; ^2^Psychology Department, University of Milano-Bicocca, Milan, Italy; ^3^Inter-University Laboratory of Human Movement Biology, Univ Lyon, University Claude Bernard Lyon 1, Villeurbanne, France; ^4^Centre for Research on Brain, Language and Music (CRBLM), Montreal, QC, Canada; ^5^Psychology Department, University of Montreal, Montreal, QC, Canada; ^6^International Laboratory for Brain, Music and Sound Research (BRAMS), Montreal, QC, Canada

**Keywords:** joint action, interpersonal synchronization, musical pleasantness, consonance, joint outcome

## Abstract

**Introduction:**

Music making is a process by which humans across cultures come together to create patterns of sounds that are aesthetically pleasing. What remains unclear is how this aesthetic outcome affects the sensorimotor interaction between participants.

**Method:**

Here we approach this question using an interpersonal sensorimotor synchronization paradigm to test whether the quality of a jointly created chord (consonant vs. dissonant) affects movement coordination. We recruited non-musician participants in dyads to perform a dyadic synchronization-continuation task (dSCT): on each trial, participants first synchronized their movements to a metronome (synchronization phase) and then continued tapping together at the same tempo without the metronome (continuation phase). Each tap yielded a note and participants heard both their own and that of their partner, thus creating a chord that was varied to be either consonant (Perf5 or Maj6) or dissonant (Min2 or Maj2). For each trial, participants also rated the pleasure they felt in creating the sounds together. Additionally, they completed questionnaires about social closeness to the other participant, musical reward sensitivity and musical training.

**Results:**

Results showed that participants' taps were closer in time when they jointly created consonant (high pleasure) vs. dissonant (low pleasure) chords, and that pleasure experienced by the dyad in each trial predicted interpersonal synchronization. However, consonance did not affect individual synchronization with the metronome or individual tapping when the metronome was discontinued. The effect of consonance on synchronization was greater in dyads who reported feeling less close prior to the task.

**Discussion:**

Together, these results highlight the role of consonance in shaping the temporal coordination of our actions with others. More broadly, this work shows that the aesthetic outcome of what we create together affects joint behaviors.

## 1 Introduction

Human cultures across the globe engage in music making: people come together and sing, play flutes, or beat drums to create aesthetically pleasing sounds. This process involves interpersonal synchronization, which entails the coordination of actions, emotions, thoughts, and even physiological rhythms among two or more people (Bernieri and Rosenthal, 1991; Ackerman and Bargh, 2010; Palumbo et al., 2017). To achieve such coordination, individuals must understand each other's intentions, adapt to different environments, take others' perspectives, and make quick decisions to synchronize effectively (Hasson et al., 2004; D'Ausilio et al., 2015; Sacheli et al., 2018). What remains unclear is the extent to which the aesthetic quality of what is jointly created affects this coordination of action.

Joint music-making provides a unique channel to study humans' ability to precisely synchronize movements in time (Repp, 2005; Repp and Su, 2013; Keller et al., 2014; Abalde et al., 2024). When playing together, musicians must adapt their production of tone sequences based on auditory information from themselves and their partners in order to synchronize effectively (Goebl and Palmer, 2009; Wing et al., 2014). Rhythmic interpersonal coordination can be measured by calculating the asynchrony between the onsets of sounds that are supposed to occur simultaneously in a piece. While some studies explored temporally precise rhythmic interpersonal coordination during naturalistic, expressive ensemble performance (Keller and Appel, 2010; Ragert et al., 2013; Keller et al., 2014; Colley et al., 2018; Laroche et al., 2022; Proksch et al., 2022), most relevant research has been conducted using sensorimotor synchronization tasks, where participants are required to perform simple movements, such as finger taps (Mates et al., 1992; Konvalinka et al., 2009, 2010; Nowicki et al., 2013; Schultz and Palmer, 2019). This task allows researchers to manipulate various conditions, providing insights into social and prosocial behaviors, as well as synchronization and cooperation processes (Konvalinka et al., 2010; D'Ausilio et al., 2015). Indeed, rhythmic joint action can be affected by factors that are related to musical expression (Keller, 2013), including tempo (Rasch, 1988; Konvalinka et al., 2010), metrical structure (Large et al., 2002; Keller and Repp, 2005; Snyder et al., 2006; Rankin et al., 2009), intensity (Goodman, 2002), and timbre (Ternström and Karna, 2002; Ternström, 2003; Sundberg, 2006). Intonation, the accurate control of pitch, is crucial for achieving consonance in music ensembles (Keller, 2013). Selective adjustments in intonation are fundamental to achieve harmonic consonance for the overall sound (Papiotis et al., 2011, 2012). However, research on interpersonal synchronization has primarily focused on the temporal aspects of coordination, neglecting the potential influence of the aesthetic quality of the joint outcome (e.g., Konvalinka et al., 2010; D'Ausilio et al., 2015).

One principal aesthetic dimension of music is consonance. Based on the work of Helmholtz (1913) and Terhardt (1984) identified sensory consonance and harmony as the two main roots of consonance. The former operates at the auditory sensation level and is linked to frequency relations, while the latter relies on pitch relationships and involves a more sophisticated cognitive process. Consonance has been investigated in literature from different perspectives, including arithmetical, psychoacoustic, neurophysiological, and cultural (for a comprehensive review, see Di Stefano et al., 2022). However, due to contrasting evidence, there is still no general consensus on how consonance is governed in music (Di Stefano et al., 2022). Nonetheless, consonant sounds are generally perceived as pleasant, while dissonant sounds as unpleasant (Trainor et al., 2002; Bendor and Wang, 2005; Di Stefano et al., 2022). This preference for consonance is observed in infants and appears to be a universal trait (Vos and Troost, 1989; Zentner and Kagan, 1998; Trainor et al., 2002; Masataka, 2006; Fritz et al., 2009).

We conjecture that the aesthetic quality of the joint outcome, particularly its consonance, might affect performance during sensorimotor tasks. One reason for this is the overlap in neural underpinnings of consonance processing and joint motor action. Specifically, Minati et al. (2009) found that consonant sounds elicited activation in the right hemisphere premotor cortex and inferior parietal lobe, among others. These brain regions are also implicated in auditory-motor integration at the individual level (Chen et al., 2008; Giovannelli et al., 2014; Lega et al., 2016; Siman-Tov et al., 2022) and in understanding others' action intentions (Ortigue et al., 2010). Based on this neural overlap, one might hypothesize that consonance influences joint action, as investigated in our study. Additionally, research with adults has shown that the learning of rules is easier when conveyed through consonant intervals compared to dissonant ones, indicating that consonance has a positive effect on higher-level cognitive abilities (Crespo-Bojorque and Toro, 2016; Di Stefano et al., 2022). To our knowledge, Komeilipoor et al. (2015) is the only study to have investigated the role of consonance in a sensorimotor synchronization task. They had individual participants perform sliding movements with their fingers to the sound of a metronome consisting of a consonant or dissonant chord. They found that consonance did not affect synchronization of movements while the metronome was present. But, puzzlingly, when the metronome was removed and participants were asked to continue moving in the same tempo, they did so less precisely and with greater variability in the dissonant (vs. consonant) condition. This result suggests that consonance has an effect on individual sensorimotor synchronization. However, it is important to recognize that in that study, participants did not themselves participate in the creation of the sound and the consonance was instead driven by an external stimulus beyond their control. Thus, it remains unclear whether sensorimotor synchronization is affected by the aesthetic quality of an individual or a jointly created outcome.

The aim of this study was to test whether the consonance of a jointly created chord affects the synchronization of movements between participants. We reasoned that when participants tap together and each person creates a sound, forming a chord, the timing of their movements would be more synchronized if the chord is consonant. We expected this effect might arise from a mutual adaptation of movement (Konvalinka et al., 2010; Nowicki et al., 2013; Van Der Steen and Keller, 2013; Keller et al., 2014; Uccelli et al., 2023), as well as from processing advantages for consonance and aesthetic pleasant chords (Bones et al., 2014; Tabas et al., 2019). If we follow this line of reasoning, we might also expect that individuals who are more sensitive to the aesthetic outcome should exhibit a greater difference between consonant and dissonant sounds. As a proxy for sensitivity to aesthetic outcome we used the extended Barcelona Music Reward Questionnaire (eBMRQ; Cardona et al., 2022). The original version of this questionnaire (BMRQ, Mas-Herrero et al., 2014) is correlated with the aesthetic facet of “Openness to Experience” section of the NEO-PI-R (Costa and McCrae, 1992), indicating that higher aesthetic sensitivity for art and beauty correlates with higher BMRQ scores. Indeed, some studies have employed the BMRQ to investigate aesthetic reward sensitivity in the music domain and beyond (Mas-Herrero et al., 2018; Hernández et al., 2019; Witek et al., 2023). Given this questionnaire's relevance for assessing aesthetic sensitivity, we used it to explore our hypothesis that those who are more sensitive to aesthetic outcome may show a greater effect of consonance. Additionally, we might expect individuals who are more socially close to show higher consonance effect. Our reasoning is that if individuals are socially close, they likely perceive themselves as part of the same group. Thus, the outcomes the outcomes of a joint action matter more than if they belonged to different groups. From an evolutionary perspective, people in the same group may have more frequent interactions with each other rather than with outsiders (“shadow of the future” effects, Axelrod, 1984). Hence, we expect that the greater the social closeness between individuals, the more they will care about the quality of their joint outcomes, resulting in a stronger impact of consonance on their interpersonal synchronization.

When testing the effect of the joint outcome on rhythmic interpersonal synchronization, it is important to take into account not only the intrinsic acoustic properties of the auditory stimulus (Goodman, 2002; Ternström and Karna, 2002; Ternström, 2003; Sundberg, 2006), but also social and psychological factors (Keller et al., 2014), and individual expertise. For instance, musical expertise is known to promote proficiency in action–effect anticipation, leading to smaller asynchronies in such interpersonal tapping task (Aschersleben and Prinz, 1995; Aschersleben, 2002; Keller and Koch, 2008; Vuust et al., 2009; Pecenka and Keller, 2011; Schultz and Palmer, 2019), and maintaining a more consistent metronome rate when receiving other feedback (Schultz and Palmer, 2019). Social skills and personality traits, such as social competence and empathy, also affect coordination timing. For example, children with higher social skills synchronize better in dyadic drumming tasks (Kleinspehn, 2008), while autistic traits are linked to deficits in interpersonal motor coordination (Curioni et al., 2017) and synchronization difficulties (Kasten et al., 2023). Investigating the effect of consonance on these aspects could provide deeper insights into how aesthetical and pleasant stimuli influences motor coordination in a population with varying levels of social skills and autistic traits. Additionally, inter-dyadic differences in spontaneous rhythm production behaviors (e.g., speech, gait, and dance) influence synchrony in joint tasks, with greater synchrony predicted by smaller differences in spontaneous production rates (Zamm et al., 2016; Palmer et al., 2019; Tranchant et al., 2022).

In the present study, we recruited non-musician participants in a dyadic synchronization-continuation task (dSCT), in which they first synchronized their movements to a metronome together (synchronization phase) and then continued tapping at the same tempo without the metronome (continuation phase). Each participant heard the auditory feedback (notes) from themselves and their partner, and we varied these sounds so that the chord they jointly created was either consonant or dissonant. If the consonance of the joint outcome and the subjective pleasure derived from it affect how paired participants synchronize their movements, we expect that consonance as well as the subjective pleasure of each chord influence the precision of the tapping coordination, both during synchronization and continuation phase (aim 1). To rule out that this effect was due to overall effects on synchronization, we also tested whether the two participants' individual synchronization to the metronome was affected by consonance (aim 2). Further, we reasoned that if the aesthetic quality of the metronome affects movement, this effect should be stronger in those who are more sensitive to the aesthetic quality of music, i.e., more sensitive to musical reward (aim 3). We also hypothesized that dyads who feel socially closer would show a greater effect of consonance on synchronization (aim 4). Finally, to confirm the validity of the measure of interpersonal synchronization, we tested whether participants who have greater musical training achieve, as expected, greater synchronization.

## 2 Method

### 2.1 Participants

Forty-two volunteers took part in the study (mean age = 23.64 ± 3.20 years; 21 men and 21 women). Participants were pseudorandomly divided into 21 dyads, ensuring they were unfamiliar with their assigned partner prior to the experiment. The dyads included seven male-male, seven female-female, and seven mixed-gender dyads (as done in Nowicki et al., 2013). All participants were neurologically healthy and did not report any hearing impairments. Most of them were right-handed (N = 38), while 4 were left-handed. Participants were non-musicians, defined as having received < 2 years of formal or informal musical training, assessed using the Musical Training subscale of the Gold-MSI questionnaire (Müllensiefen et al., 2014). The experimental protocol was approved by the local ethics committee of the University of Pavia (Ethical Committee Prot. # 132/23) and participants were treated in accordance with the Declaration of Helsinki.

### 2.2 Materials

**Figure 1:**
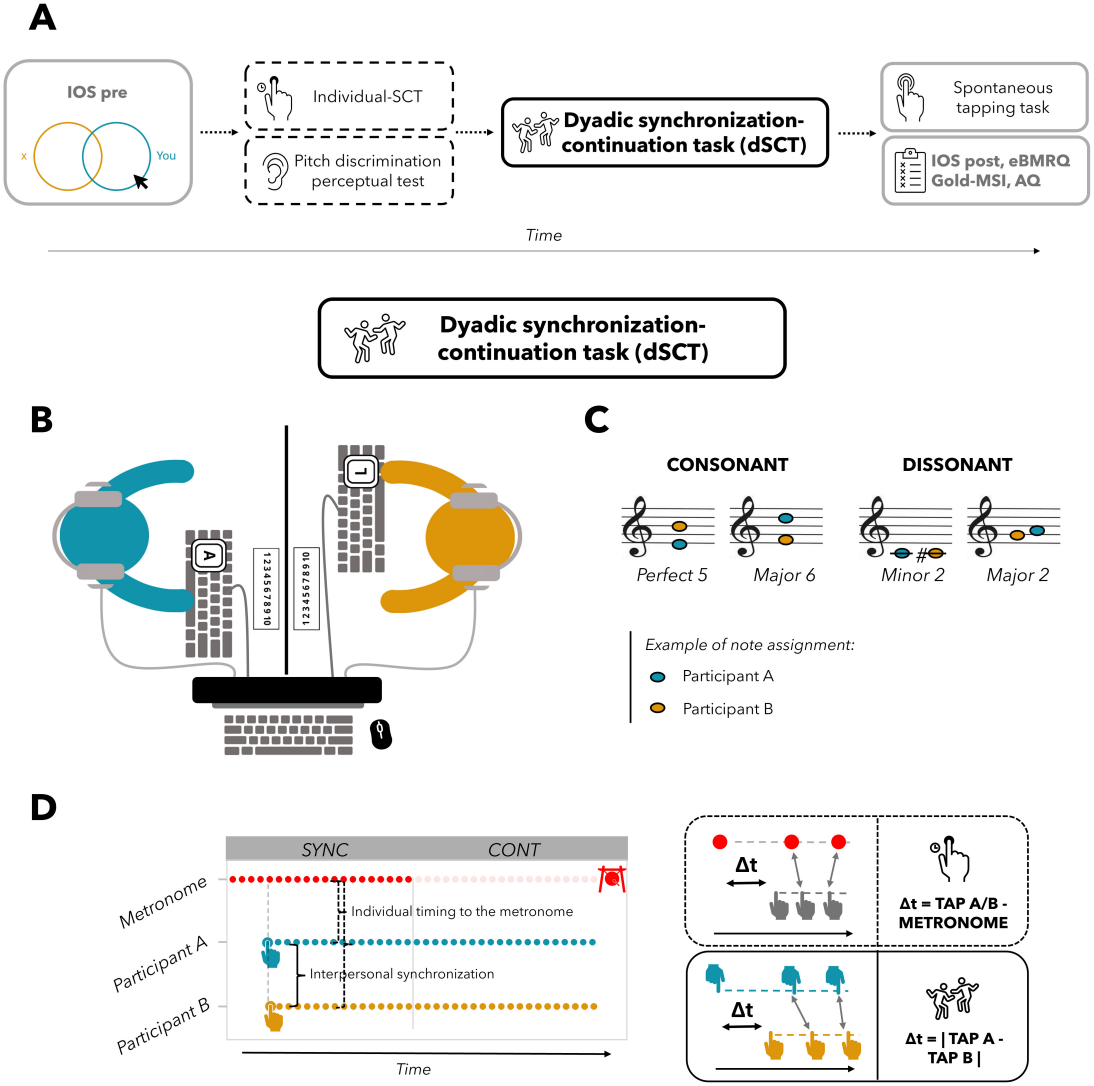
**(A)** Flow chart of the procedure. IOS, Inclusion of Other in Self scale, administered once before (pre) and once after (post) the dSCT; Individual-SCT, individual synchronization-continuation task; dSCT, dyadic synchronization-continuation task; eBMRQ, extended Barcelona Music Reward Questionnaire; Gold-MSI, Goldsmith Musical Sophistication Index; AQ, Autism Quotient Questionnaire. Assessment of inter-individual differences is outlined in gray, while the assessment of pitch discrimination and synchronization abilities with a dashed line. **(B)** Top view of the experimental setup. Participants sat facing each other on opposite sides of the table, with a central panel preventing them from seeing each other; they wore noise-canceling earphones. Participant A of the dyad (in blue) taps on the “A” key of one keyboard, while participant B (in yellow) on the “L” key of the other. Keyboards and earphones were linked to the same computer. **(C)** The four musical chords employed in the experiment, divided into consonant and dissonant ones. **(D)** Dyadic synchronization-continuation task (dSCT) structure. Red dots indicate clicks of the metronome, which is discontinued in the continuation phase, while blue (Participant A) and yellow (Participant B) dots refer to participants' taps. A gong (depicted on top right corner) indicates the end of each trial. Time differences between each participant's tap and the closest metronome click (Δt = tap A/B – metronome) were calculated for the individual timing in the synchronization phase, while time differences between participant A and B closest taps were computed for the interpersonal synchronization (Δt = |tap A – tap B|) in both synchronization and continuation phase, bottom right corner of the Figure.

The whole experimental procedure is charted in Figure 1A. With regard to the dyadic synchronization task, participants were positioned facing each other on opposite sides of the table, with a panel placed in the center to obstruct their view of the other participant during the task (Figure 1B). Each participant tapped on a computer keyboard placed in front of them. The two keyboards used by participants in the dyad were linked to the same computer. During the task, one participant pressed the A key on one keyboard while the other pressed the L key on the other keyboard. To receive the sound feedback as well as to ensure participants could not hear the sounds produced by clicking the keys, they were equipped with noise-canceling earphones (see Figure 1B). Taps were recorded by a custom Python interface running Pygame (a set of bindings to Simple DirectMedia Layer, SDL, connected to the two keyboards), which also created the sound of feedback and the click of the metronome, as well as the final gong indicating the end of each trial (see Figure 1D). The metronome sound was a woodblock sound wave file of 30 ms duration, included by default in the Teensy Python interface (Van Vugt, 2020; see also Schultz and Van Vugt, 2016), while the duration of the tap feedback sound was either 150, 200, or 400 ms (held constant within each trial). Each participant received auditory feedback in the form of one of eight distinct tones synthesized as pure sine waves with a 5 ms linear fade in and one of the following frequencies: C (261 Hz), C# (277.18 Hz), E (329.63 Hz), F (349.23 Hz), G (392 Hz), A (440 Hz), B (493.88 Hz), and D (587.33 Hz). During the dyadic synchronization-continuation task (see details below), each participant heard the auditory feedback from themselves and the other, thus creating a chord, which could be either consonant (Perf5 or Maj6) or dissonant (Min2 or Maj2). We use “chord” here to refer to two notes played simultaneously, as shown in Table 1 and Figure 1C. The selection of these chords was based on previous studies (Krumhansl and Cuddy, 2010; McDermott et al., 2010). These studies revealed that Perf5 and Maj6 chords received high pleasure ratings. Conversely, Min2 and Maj2 chords were associated with low ratings of pleasure. These results were obtained when participants rated chords from very unpleasant to very pleasant (McDermott et al., 2010), as well as when indicated how effectively a tone completed an unfinished scale, such as how well the C note concluded the ascending scale C-D-E-F-G-A-B (from very badly to very well, Krumhansl and Cuddy, 2010). The onset of each tap of both participants as well as metronome timings were written to a file for offline analysis (see Van Vugt, 2020).

**Table 1:** Auditory stimuli employed in the experiment.

	**Note assigned to participant A of the dyad**	**Note assigned to participant B of the dyad**	**Chord**	**Nominal consonance**
1	C	C#	Min2	Dissonant
2	C#	C	Min2	Dissonant
3	G	A	Maj2	Dissonant
4	A	G	Maj2	Dissonant
5	E	B	Perf5	Consonant
6	B	E	Perf5	Consonant
7	D	F	Maj6	Consonant
8	F	D	Maj6	Consonant

### 2.3 Procedure

After participants arrived in the lab, they responded to questionnaires, underwent perceptual testing, and performed individual and joint tapping tasks, as illustrated in Figure 1A. Comprehensively, the procedure lasted 2 h.

#### 2.3.1 Perceived social closeness

Participants were asked to indicate the perceived social closeness to the other participant of the dyad using the *Inclusion of Self in the Other scale* (IOS; Aron et al., 1992), implemented via the jsPsych plugin (see Kinley and Van Vugt, 2023). Participants were asked to indicate their perceived closeness to their partner in the dyad by adjusting the amount of overlap of two circles (see example in Figure 1A), where greater overlap indicated higher perceived closeness. This test was conducted both before (pre) and after (post) the collaborative tasks to measure changes in closeness (see Figure 1A). We are interested in the effects of previously existing closeness, uncontaminated by changes that might happen as a result of the experiment, on the interpersonal synchronization and on the consonance effects. Additionally, we aim to look at the changes between the two measures' timing. To mitigate the influence of social desirability bias, participants completed the test privately on both occasions, ensuring their responses remained undisclosed to their pairs.

#### 2.3.2 Assessment of pitch discrimination and synchronization abilities

We reasoned that two prerequisites for adequately performing the dyadic synchronization-continuation task (dSCT) are I) the perceptual ability to discriminate between the chords used in the experiment and II) the ability to motorically synchronize with the metronome. Thus, prior to the dSCT, participants performed two preliminary tasks, evaluating pitch discrimination (*pitch discrimination perceptual test*) and sensorimotor synchronization abilities (*individual synchronization-continuation task, SCT*; see Figure 1A). The *pitch discrimination perceptual test* aimed to assess that participants could accurately distinguish between the chords used in the dSCT. Participants listened to a total of 10 chord pairings, consisting of combinations of the chords listed in Table 1, thus creating 6 pairings with different and 4 with the same chords. Participants were required to indicate whether chords in each pair were identical or different by pressing either the A or L key on the computer (counterbalanced across participants). The software OpenSesame (Mathôt et al., 2012) was used for stimuli presentation and data collection. The individual sensorimotor synchronization abilities of each participant of the dyad were assessed through an *individual-SCT*. In this task, participants were instructed to synchronize their tapping with the metronome (synchronization phase), starting at the fifth click, using their dominant hand on the assigned key (A or L, counterbalanced across participants). After this phase, consisting of 20 metronome clicks, the metronome stopped, and participants were told to continue tapping for a duration equivalent to 20 clicks, maintaining the same tempo (continuation phase). At each tap, participants received auditory feedback in the form of A note (440 Hz). Each trial concluded with the sound of a gong. Participants underwent a total of nine trials, determined by the random combination of three metronome tempo (Inter-Onset Intervals, IOI; 450, 550, or 650 ms) x 3 auditory feedback durations (150, 200, and 400 ms).

#### 2.3.3 Dyadic synchronization-continuation task

The materials and the procedure employed in this task are similar to the individual-SCT. Participants were instructed not only to synchronize their taps with the metronome, as they did in the individual-SCT, but also to align their taps with each other. While tapping, participants received auditory feedback (i.e., a note) from both themselves and their partner. If they tapped simultaneously, they jointly created a chord, which could be either consonant or dissonant (see Figure 1C). Based on which note was assigned to each participant in each trial, the dyad could create a total of eight different chords (refer to Table 1). These instructions were chosen so that they could apply to both the synchronization and continuation phases equally. The dyads completed 72 trials, which were randomly determined by combining three metronome IOI (450, 550, or 650 ms), three auditory feedback durations (150, 200, and 400 ms) and eight chords (see Table 1). A break was offered when half of the trials were completed. At the end of each trial, participants were asked to rate how much they liked the chord they produced together on that trial (*subjective ratings of pleasure*) on a scale from 1 to 10. We instructed participants to consider this range from very unpleasant to highly pleasant sounds, to use the entire rating scale, and to rate independently of their synchronization with the other. To provide their ratings, participants indicated with their hand the chosen number on a paper sheet, hidden from the view of the other (Figure 1B).

#### 2.3.4 Individual spontaneous tapping rate

Each participant engaged in a *spontaneous tapping task* to assess their spontaneous tapping rate individually without a pacing stimulus (see Figure 1A). Participants A and B of each dyad performed this task separately. They were asked to tap as regularly as possible for about 1 min at a comfortable, self-chosen pace (Wing and Kristofferson, 1973; Hammerschmidt et al., 2021; Pfordresher et al., 2021), while the other participant waited. This test aimed to be able to control for spontaneous tapping rates in joint synchronization tasks (see Zamm et al., 2016; Tranchant et al., 2022). Since this analysis was not directly relevant to our aims the results are included in the Supplementary Section 4.

#### 2.3.5 Music reward sensitivity

Furthermore, participants completed the *extended* version of the *Barcelona Music Reward Questionnaire* (eBMRQ; Cardona et al., 2022) to measure their music reward sensitivity. This questionnaire consists of 24 items, divided into six subscales: Music Seeking, Emotion Evocation, Mood Regulation, Sensorimotor, Social, and Musical Absorption, with four items per subscale. Each item (e.g., “When I share music with someone, I feel a special connection with that person.”) requires responses on a 5-point Likert scale, ranging from “completely disagree” to “completely agree.”

#### 2.3.6 Musical training and perceptual abilities

Then, each participant filled out the *Musical Training* and *Perceptual Abilities* subscales of the *Goldsmith Musical Sophistication Index* (Gold-MSI; Müllensiefen et al., 2014) to evaluate the influence of musical expertise on interpersonal tapping abilities. The Musical Training subscale comprises 7 items, such as “I engaged in regular, daily practice of a musical instrument (including voice) for N years,” with N varying across a 7-point scale for each item (e.g., 0, 1, 2, 3, 4–5, 6–9, 10+). Responses are then scored from 1 to 7, based on the position of the number of years within the scale (for instance, 0 years is scored as 1, 1 year as 2, 2 as 3, and so on, up to 10+ scored as 7). The *Perceptual Abilities* subscale includes 9 items (e.g., “I am able to judge whether someone is a good singer or not”) and require responses on a 7-point Likert scale, ranging from “totally disagree” to “totally agree.”

#### 2.3.7 Autism

Lastly, participants completed the *Autism Quotient Questionnaire* (AQ; Ashwood et al., 2016) to investigate autistic-like traits influence (Tryfon et al., 2017; Bloch et al., 2019; Granner-Shuman et al., 2021; Carnevali et al., 2024) on sensorimotor synchronization abilities. This 50-item questionnaire offers four response options, ranging from “totally agree” to “totally disagree.” For some items, points are given for disagreeing (e.g., “I prefer to do things with others rather than on my own”), while in others for agreeing (e.g., “I prefer to do things the same way over and over again”).

### 2.4 Data analysis

#### 2.4.1 Assessment of pitch discrimination and synchronization abilities

In the *pitch discrimination perceptual test*, we computed the mean and standard deviation of correct responses (out of ten) to verify participants' ability to distinguish sounds. Additionally, this task enabled us to screen participants for amusia (Peretz et al., 2002; Liu et al., 2017; Whiteford and Oxenham, 2017). We observed a mean of 8.95 correct responses, with a standard deviation of 1.03. All participants performed above chance level (X = 5), as indicated by the significant one-sample *t*-test against the hypothesis μ = 5 [*t*_(41)_ = 24.75, *p* < 0.001], confirming adequate ability in distinguishing between the chosen chords. We then analyze the *individual synchronization-continuation task (SCT)* as follows: we computed the signed timing difference between each tap and the nearest metronome click (in ms) and then we aggregated these differences within each of the nine trials for each participant, to determine the mean and SD (in ms). The distribution of the means and SDs across participants had the following parameters: Skewness_M_ = 0.08, Kurtosis_M_ = 3.72; Skewness_SD_ = 0.84, Kurtosis_SD_ = 2.42. The mean values ranged from a minimum of −181.90 ms to a maximum of 225.81 ms. As a result, we determined that all participants have normal proficiency in both pitch discrimination and sensorimotor synchronization abilities. Consequently, we decided to retain the entire sample for further analysis.

#### 2.4.2 Dyadic synchronization-continuation task

We analyzed the *subjective ratings of pleasure* performing a within-participants ANOVA with consonance of the chords (two levels: consonant vs. dissonant), auditory feedback duration (three levels: 150, 200, and 400 ms) and metronome tempo (three levels of IOI: 450, 550, and 650 ms) as factors, to assess whether the consonant chords were rated higher than dissonant ones (see Krumhansl and Cuddy, 2010; McDermott et al., 2010), as well as to see differences in pleasure rating based on the duration of the sound and the metronome tempo. Then, we analyzed tapping data inspecting *interpersonal synchronization* and *individual tapping precision*. Both synchronization and continuation phases were included in the analyses. For *interpersonal synchronization*, we analyzed both phases (synchronization and continuation) in the same way: by measuring the time difference between the taps of the two participants. We incorporated the factor “Task Phase” in the ANOVA model. For *individual tapping precision*, we conducted different analyses for the two phases: during the synchronization phase, we analyzed the time difference between the participant's taps and the metronome, while for the continuation phase, when the metronome was discontinued, we examined the Inter-Tap Intervals (ITIs), Thus, we performed two different ANOVA models, one for each phase. Specifically, when investigating participants' *interpersonal synchronization*, we firstly excluded a few trials (*n* = 6, 0.39% of all trials) in which, due to a technical glitch, only one participant's taps were recorded. Then, we paired each tap from participant A with the tap from participant B that was closest in time, and we calculated the absolute time difference (in ms) between the two taps (Δt = |tap A – tap B|; see Figure 1D). We excluded taps after the end-of-trial sound signal and, to avoid incorrect tap matching (e.g., participant B started tapping later compared to participant A, thus the dyad has not started synchronizing yet), we also removed absolute difference values > 80%^*^metronome IOI. Following this criterion, 1.05% of matching pairs were excluded. We aggregated these absolute taps differences within each of the 72 trials for each dyad to determine the mean of absolute tap time difference (in ms), both for the synchronization and the continuation phase, using the formula *mean(*Δ*t)*, where Δt is the time difference calculated above. The distribution of the means of absolute taps difference showed considerable departure from normality (Skewness = 2.36, Kurtosis = 10.73), so we applied a logarithmic transformation (transformed scores Skewness = 0.55, Kurtosis = 3.17). These log transformed means, calculated within each trial, were then averaged across the trials of the same dyad, separately for each task phase, tempo, auditory feedback duration and consonance of the sound (36 values per dyad). After this data pre-processing, we performed a within-dyads ANOVA on log transformed means with consonance of the chords (two levels: consonant vs. dissonant), tempo (three levels of metronome IOI: 450, 550, and 650 ms), auditory feedback duration (three levels: 150, 200, and 400 ms) and task phase (two levels: synchronization vs. continuation) as factors.

To examine the *individual tapping precision* and determine whether this measure is affected by consonance, for the synchronization phase we analyzed each individual's timing deviation from the metronome. We matched each tap with the closest (in time) click of the metronome, and we calculated the signed time difference (in ms) between the metronome click and the nearest participant's tap for this phase (Δt = tap A/B – metronome; see Figure 1D). We then calculated the mean and variability of these signed time differences (in ms), with the formulas *mean(*Δ*t)* and *sd(*Δ*t)*. Due to the presence of negative values and the adherence to the normality assumption for the means' and SDs' distributions (Skewness_M_ = −0.17, Kurtosis_M_ = 3.39, Skewness_SD_ = 1.09, Kurtosis_SD_ = 2.95), we opted not to apply a logarithmic transformation to the variables. Consequently, the values will be reported in their original scale. The means and SDs of signed time differences calculated within each trial were then averaged across the trials of the same participant (18 values per participant). Thus, we performed two within-participant ANOVAs with mean and variability (SD) of signed time differences as dependent variables, and the same set of variables described above as factors, except for task phase. For the continuation phase, where the metronome is discontinued, we analyzed the consonance effect on the Inter-Tap Intervals (ITIs). We calculated ITIs between consecutive taps of the same participant, and then averaged them across trials to determine the mean and standard deviation of ITIs (18 values per participant). Thus, we performed two within-participant ANOVAs with mean ITI and standard deviation ITI as the dependent variables (in ms), and the same set of factors described above.

All the ANOVA models were performed using the *ez* package (Lawrence, 2016) in the R statistical language (R Core Team, 2023). *Post-hoc* comparisons were computed using the package *rstatix* (Kassambara, 2019) with Holm correction method. Following the recommendation of Bakeman (2005), we reported generalized effect sizes ($\eta_{\text{G}}^{2}$; Olejnik and Algina, 2003).

Lastly, regardless of the consonant/dissonant properties of the auditory stimuli, we investigated if dyadic pleasure influences how participants synchronize their movements (*pleasure and interpersonal synchronization relationship*). For each dyad, we collected all trials, and we computed a per-dyad regression slope between pleasure (calculated as the average score between participant A and participant B for each trial) and the mean absolute tap differences (in log ms). We then tested whether these regression slopes were significantly different from zero on the group level using a *t*-test.

#### 2.4.3 Assessment of psychological constructs

We calculated the difference between post-experimental and pre-experimental closeness (IOS) scores for each dyad, hence yielding a change in closeness rating. For clarity, we expressed IOS scores as a percentage. To examine whether the effect of consonance correlates with closeness or musical reward sensitivity, we first averaged the mean absolute tap differences (in log ms) for both consonant and dissonant trials for each dyad and we calculated an estimate of the dyad's consonance effect by subtracting the mean dissonant score from the mean consonant score for each dyad. Then we computed the correlation between this dyad consonant effect and the dyad-summed musical reward and closeness scores (two separate correlations).

Additionally, we conducted an exploratory analysis investigating whether dyadic differences in social factors (i.e., social closeness and autism), musical experience and music reward sensitivity correlate with interpersonal synchronization. We summed the scores of participant A and participant B for each questionnaire (IOS pre, eBMRQ, AQ, Musical Training, and Perceptual Abilities) and we employed these dyadic scores sum in a correlation analysis with the mean of absolute taps difference (in log ms) aggregated for each dyad. We performed the correlation analyses using package *stats* in the R statistical language (R Core Team, 2023).

## 3 Results

### 3.1 Dyadic synchronization-continuation task

#### 3.1.1 Subjective rating of pleasure

**Figure 2:**
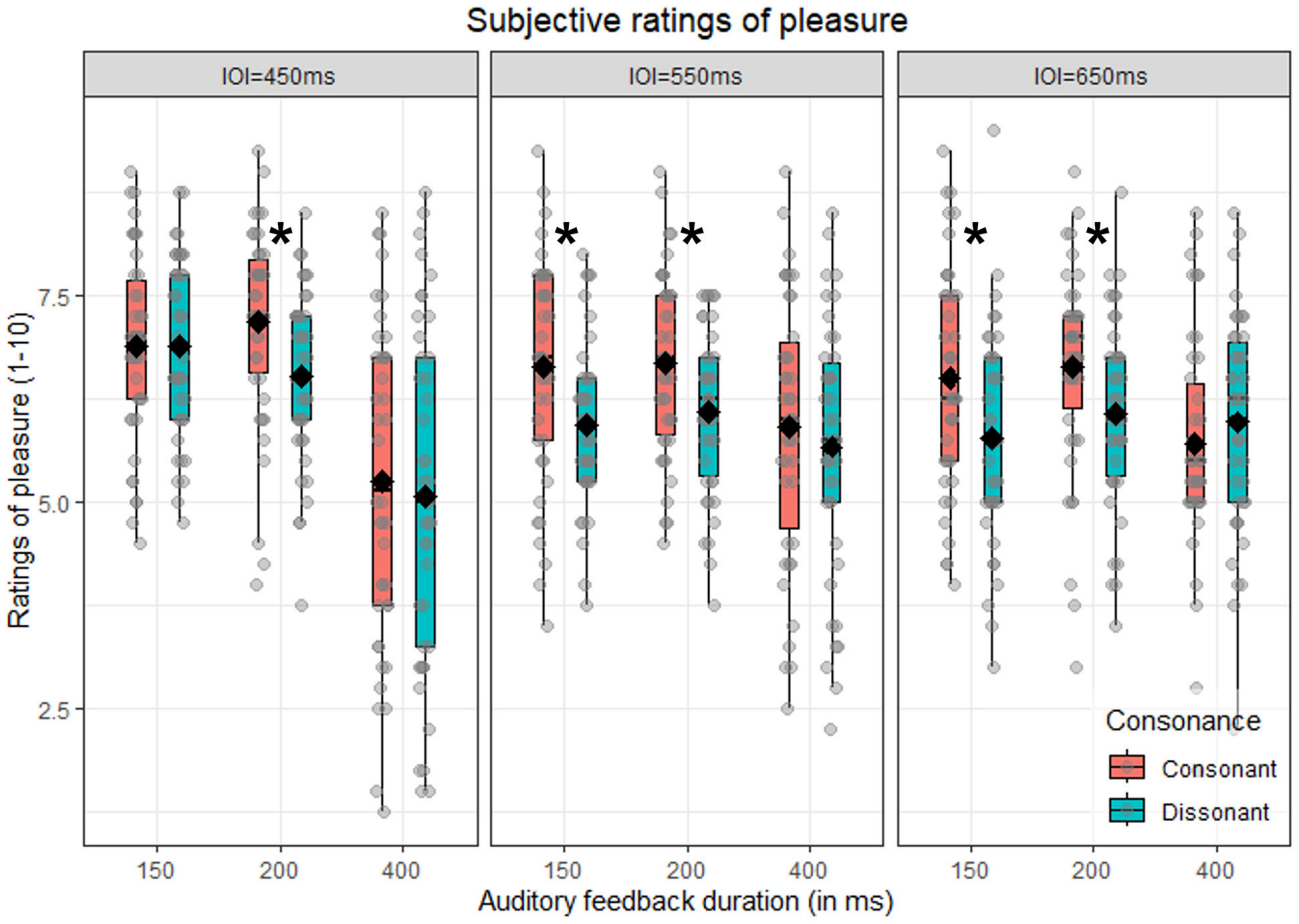
Subjective rating of pleasure. Boxplot of the subjective ratings of pleasure as a function of auditory feedback duration (150, 200, and 400 ms), consonance (consonant vs. dissonant) and split by metronome IOI (450, 550, and 650 ms). Diamonds indicate the mean for each condition, while dots refer to the single participants' mean pleasure rating for that specific combination of conditions. Asterisks indicate a significant difference between consonant vs. dissonant in that specific combination of conditions.

The main aim of this analysis is to confirm that subjective pleasure is predicted by consonance. Additionally, we investigated whether this effect interacted with both feedback duration and metronome IOI. Thus, we performed a repeated-measure ANOVA with consonance, auditory feedback duration and metronome IOI as within-subjects factors. As expected, we found a significant main effect of consonance [*F*_(1, 41)_ = 7.61, *p* = 0.009, $\eta_{\text{G}}^{2}$ = 0.02]: consonant chords were rated significantly higher (M = 6.37, SD = 1.50) compared to dissonant ones (M = 6.00, SD = 1.39), as illustrated in **Figure 4A**. This effect significantly interacted with both feedback duration and metronome IOI, as indicated by the significant three-way interaction consonance x metronome IOI x auditory feedback duration [*F*_(4, 164)_ = 2.77, *p* = 0.029, $\eta_{\text{G}}^{2}$ = 0.01]. To further explore this interaction, we analyzed pleasure ratings separately by metronome IOI and auditory feedback duration, to test in which combination of conditions consonant chords were rated higher than dissonant ones. This was true for the IOI = 550 ms [*t*_(41)_ = 2.97, *p* = 0.028] and IOI = 650 ms [*t*_(41)_ = 3.80, *p* = 0.004] in the 150 ms auditory feedback duration, and for all the metronome IOI conditions in the 200 ms auditory feedback duration condition [450 ms: *t*_(41)_ = 3.50, *p* = 0.008; 550 ms: *t*_(41)_ = 3.03, *p* = 0.028; 650 ms: *t*_(41)_ = 3.05, *p* = 0.028], as illustrated in Figure 2. In sum, consonant trials are overall associated with higher pleasure (main consonance effect), although the pattern is not significant across all combinations of metronome tempo and auditory feedback duration (interaction). Since the main interest of this analysis was the effect of consonance on subjective pleasure, the interactions that do not involve consonance have been moved to Supplementary Section 1.

#### 3.1.2 Interpersonal synchronization (dSCT)

To test whether consonance affected interpersonal synchronization (aim 1), we performed an ANOVA on the interpersonal synchronization (calculated as mean of absolute taps difference in log ms), which revealed a main significant effect of consonance [*F*_(1, 20)_ = 7.99, *p* = 0.010, $\eta_{\text{G}}^{2}$ = 0.01]. Crucially, participants demonstrated better synchronization with each other when they produced a consonant chord (3.73 log ms, 48.55 ms) compared to a dissonant one (3.78 log ms, 51.89 ms), as illustrated in Figure 4B. None of the interaction effects with consonance were significant (all *Fs* < 2.37, *ps* > 0.107). Since the main interest of our study was on the effect of consonance, all the other main or interaction effects that do not involve consonance have been moved to Supplementary Section 2.

#### 3.1.3 Pleasure and interpersonal synchronization

**Figure 3:**
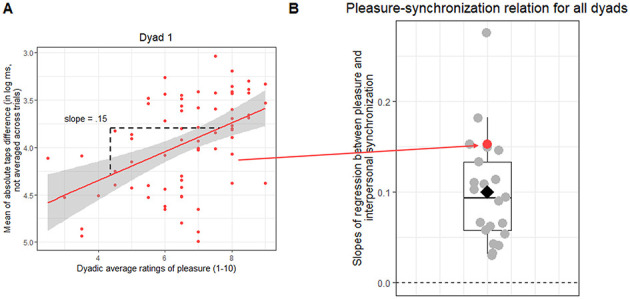
Pleasure and interpersonal synchronization relationship for all dyads. **(A)** Shows one example dyad. Each dot corresponds to one of the 72 trials. The red line indicates the regression model fit, predicting the mean of absolute taps difference (in log ms, not averaged per trial) by dyadic average ratings of pleasure (slope = 0.15), and the shaded area its standard error. Note that the y axis is inverted with higher values suggesting better interpersonal synchrony. The slope was extracted for group analysis. This analysis was repeated for all dyads individually. **(B)** Shows the slopes for all the dyads as dots. The red dot indicates the slope for the dyad shown in **(A)**; the diamond indicates the slopes' mean. Higher values indicate steeper slope lines.

When analyzing the correlation between dyadic pleasure rating and interpersonal synchronization (aim 1), we found a positive slope in all dyads (mean = 0.10, SD = 0.06), indicating that the higher the pleasure, the higher is the interpersonal synchronization. This slope was significantly different from zero on the group level [*t*_(20)_ = 7.78, *p* < 0.001; Figure 3B]. We conclude that, independently from the metronome IOI and the auditory feedback duration, dyads tended to tap more closely together on trials that were rated as more pleasant (see Figures 3A, B).

#### 3.1.4 Individual tapping precision (dSCT)

**Figure 4:**
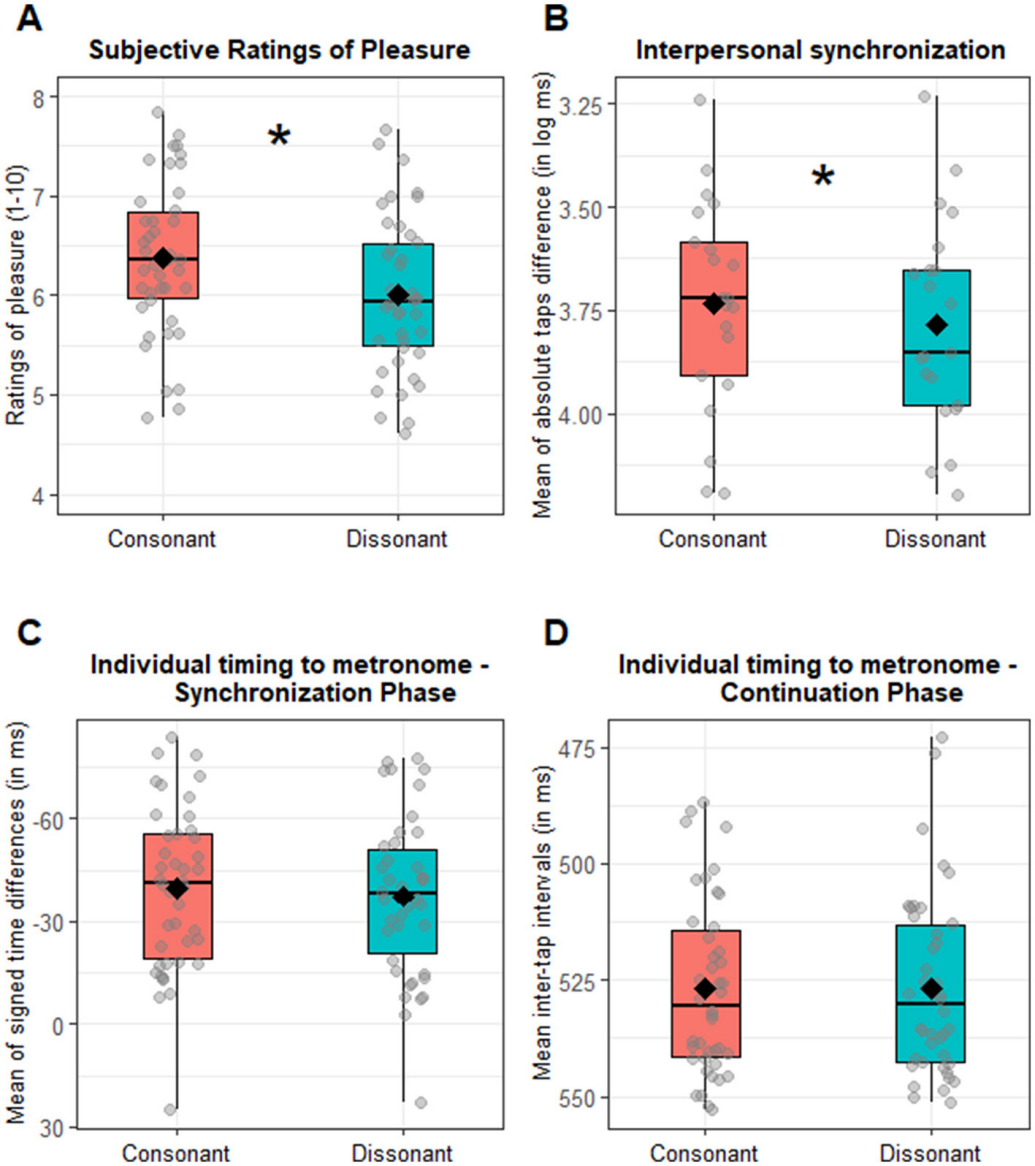
Consonance effect. **(A)** Boxplot of the subjective ratings of pleasure as a function of consonance (consonant vs. dissonant). Dots refer to the individual participants' mean rating of pleasure for each condition. **(B)** Boxplot of consonance effect on interpersonal synchronization (measured as the mean of absolute taps difference log transformed). Diamonds indicate the general consonant and dissonant means, while dots refer to the dyadic mean of absolute taps difference for each condition. Note that the y axis is inverted so that higher values suggest better interpersonal synchrony. **(C)** Boxplot of the consonance effect on the individual tapping precision (tap timing relative to the metronome) in the synchronization phase (measured as the mean of signed time differences in ms). Diamonds indicate the general consonant and dissonant means, while dots refer to the individual mean of signed difference for each condition. Zero suggests perfect synchronization with the metronome. **(D)** Boxplot of the consonance effect on individual tapping precision in the continuation phase (measured as mean inter-tap interval, mean ITI). Diamonds indicate consonant and dissonant mean ITI, while dots refer to the individual mean ITI for each condition. Asterisks indicate a significant difference between consonant vs. dissonant condition.

To test whether consonance affected individual timing relative to metronome (aim 2), we performed an ANOVA on the mean of signed time differences (in ms) during the synchronization phase. No significant main effect of consonance emerged [*F*_(1, 41)_ = 2.42, *p* = 0.127, $\eta_{\text{G}}^{2}$ = 0.003; M_Consonant_ = −39.53 ms; M_Dissonant_ = −37.37 ms], as shown in Figure 4C. None of the other interaction effects with consonance reached significance either (all *Fs* < 1.65, *ps* > 0.164). Additionally, we performed the same analysis on the variability (SD) of signed time differences (in ms). This analysis indicated again no significant main effect of consonance [*F*_(1, 41)_ = 3.32, *p* = 0.076, $\eta_{\text{G}}^{2}$ = 0.003; SD_Consonant_ = 63.47 ms; SD_Dissonant_ = 61.06 ms]. None of the interaction effects with consonance reached the significance (all *Fs* < 1.61, *ps* > 0.173). Since the main interest of our study was on the effect of consonance, the interaction effects not involving consonance have been included in Supplementary Section 3A. In the continuation phase, when investigating the effect of consonance, the ANOVA on the mean inter-tap interval (ITIs) indicated no significant main effect of consonance [*F*_(1, 41)_ = 0.07, *p* = 0.789, $\eta_{\text{G}}^{2}$ < 0.001; M_Consonant_ = 525.72 ms; M_Dissonant_ = 525.94 ms], see Figure 4D. The interaction between auditory feedback duration and consonance [*F*_(2, 82)_ = 3.83, *p* = 0.026, $\eta_{\text{G}}^{2}$ = 0.01] was statistically significant. However, corrected *post-hoc* comparisons testing for consonance vs. dissonance differences within each auditory feedback duration did not reveal any significant differences [all *ts*_(41)_ < 2.15, *ps* > 0.114; Supplementary Section 3B, Supplementary Figure 3B]. No other interactions with consonance were significant (all *Fs* < 1.62, *ps* > 0.173). Looking at the variability (SD) in the ITIs as a function of consonance, the ANOVA revealed only a trend toward a significant consonance effect [*F*_(1, 41)_ = 3.81, *p* = 0.058, $\eta_{\text{G}}^{2}$ =.003; SD_Consonant_ = 30.88 ms; SD_Dissonant_ = 32.30 ms]. None of the interaction effects with consonance reached significance (all *Fs* < 1.47, *ps* > 0.213). Since the main interest of our study was on the effect of consonance, the interaction effects not involving consonance have been included in Supplementary Section 3B. In sum, we found no overall significant effects of consonance on individual tapping performance, neither in terms of synchronization to the metronome nor in terms of tapping continuation without the metronome.

### 3.2 Assessment of psychological constructs

Table 2 reports the general mean, SD and maximum value for each questionnaire. IOS values, expressed as a percentage, were significantly higher after the experiment than before [*t*_(41)_ = −4.26, *p* < 0.001], demonstrating an increased perceived closeness after the experiment.

**Table 2:** Mean, SD, and maximum value for each questionnaire.

	**General mean**	**SD**	**Questionnaire maximum value**
Closeness (IOS pre)	33.8%	32.5%	100%
Closeness (IOS post)	51.4%	29.2%	100%
Closeness change (post—pre IOS)	17.6%	26.8%	100%
Music reward sensitivity (eBMRQ)	91.2	11.3	120
Musical training (Gold-MSI)	13.1	7.01	49
Perceptual abilities (Gold-MSI)	45.4	6.53	63
Autism quotient (AQ)	14.9	6.06	50

#### 3.2.1 Inter-dyadic differences on consonance effect

**Figure 5:**
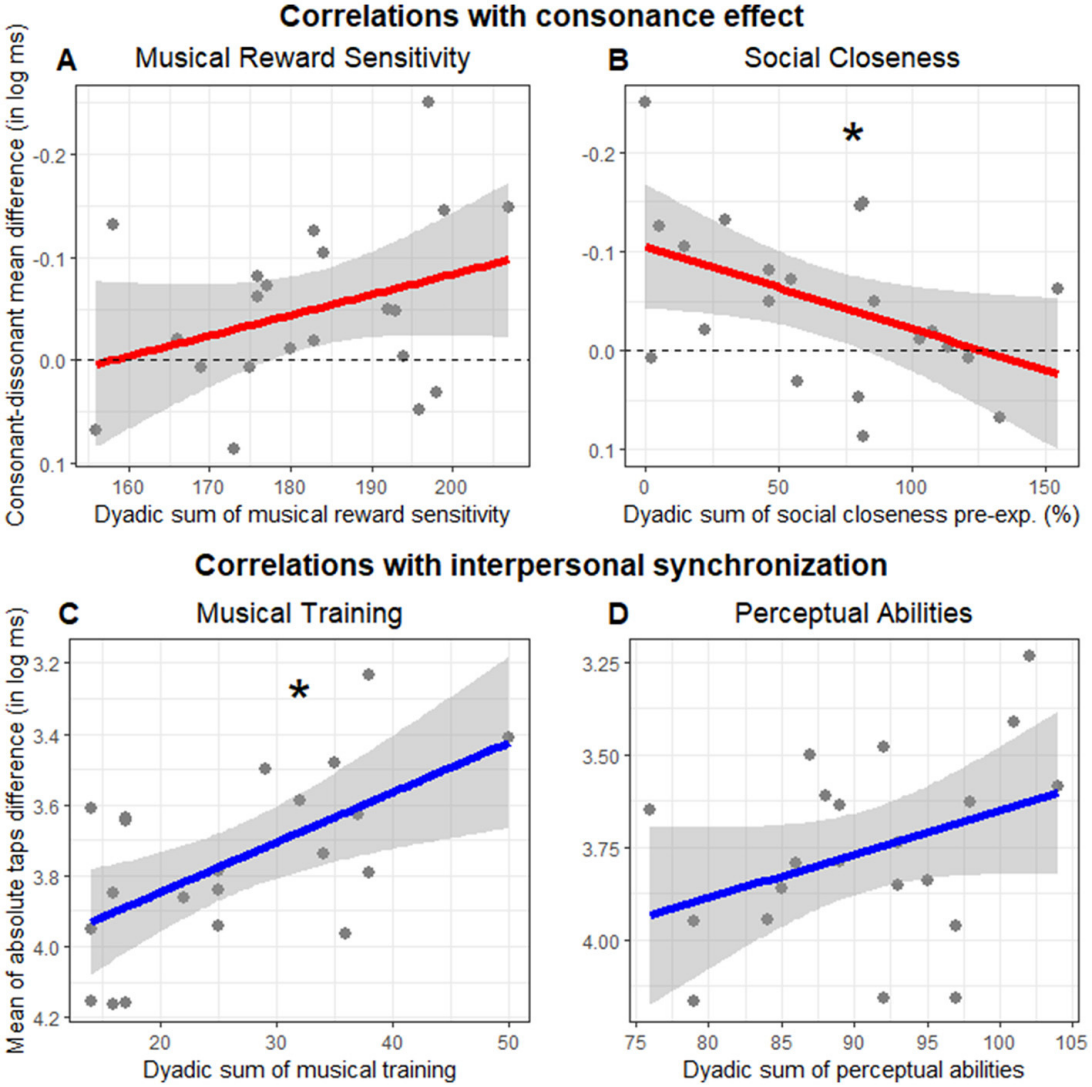
Correlation with consonance effect **(A, B)** and interpersonal synchronization **(C, D)**. **(A, B)** Depict the consonance effect (measured as interpersonal synchronization in the consonant minus the dissonant trials, in log ms) by dyadic sum of **(A)** music reward sensitivity (eBMRQ) and **(B)** social closeness before the experimental session (IOS pre, expressed in percentage). Higher points suggest higher consonant effect. Dashed lines indicate values with no consonance-dissonance difference. **(C, D)** Represent the interpersonal synchronization (measured as the mean of absolute taps difference in log ms) as a function of the dyadic sum of **(C)** musical training and **(D)** perceptual abilities. Each point indicates the mean of absolute taps difference for each dyad. Note that the y axis is inverted with higher values suggesting better interpersonal synchrony. Lines indicate the regression model fit and the shaded area its standard error. Asterisks indicate significant correlations.

To investigate the effect of musical reward sensitivity (aim 3) and perceived closeness (aim 4) on consonance, we performed a correlation analysis between the consonance effect (calculated for each dyad as the interpersonal synchronization in the consonant minus the dissonant trials) and both eBMRQ dyadic scoring sum and IOS pre dyadic sum. The dyadic music reward sensitivity scores did not significantly correlate with the consonance effect (*r* = −0.33, *p* = 0.139; Figure 5A). The perceived closeness, uncontaminated by changes that might happen as a result of the experiment (IOS pre), significantly correlated with the consonance effect (*r* = 0.46, *p* = 0.038), but the direction of this effect was opposite to what we had hypothesized: the higher the perceived closeness before the experiment, the *smaller* the effect of consonance during dSCT (Figure 5B). Since we did not have hypotheses about the relationships between the consonance effect and the other questionnaire scores, we have moved these to the Supplementary Section 6A.

#### 3.2.2 Inter-dyadic differences on interpersonal synchronization

We performed an exploratory correlation analysis to investigate the relationships between dyadic scoring sum of each questionnaire and interpersonal synchronization (measured as the mean of absolute taps difference in log ms). Only the dyadic musical training sum showed a significant correlation with interpersonal synchronization (*r* = −0.59, *p* = 0.005, Figure 5C). In contrast, the correlation with perceptual abilities was not significant (*r* = −0.36, *p* = 0.105, Figure 5D). These results indicated that the higher the dyadic musical training sum, the higher their interpersonal synchronization. Social factors (IOS pre: *r* = −0.04, *p* = 0.864; AQ: *r* = −0.27, *p* = 0.232), as well as eBMRQ dyadic scores sum (*r* = −0.19, *p* = 0.415) did not correlate significantly with interpersonal synchronization. We reported all the correlations between questionnaire dyadic scoring sum and interpersonal synchronization in Supplementary Section 6B.

## 4 Discussion

The present study investigated whether the quality of a joint outcome can shape the dynamics of interpersonal movement synchronization between individuals. Paired non-musician participants performed a dyadic synchronization–continuation task (dSCT). Each participant heard the auditory feedback from themselves and their partner, thus creating a chord, which could be either consonant (Perf5 or Maj6) or dissonant (Min2 or Maj2). Results showed that interpersonal synchronization accuracy was higher when participants produced consonant chords together (high pleasure), compared to dissonant ones (low pleasure). Since the consonant and dissonant conditions (varied within dyads) only differed in the pitch content, with no differences in auditory feedback duration and metronome tempo, we argue that the interpersonal sensorimotor timing differences observed are driven by the consonance created by the dyad. Supporting this finding, we also found that the dyad's subjective rating of pleasure from the chord they produced together predicted interpersonal synchronization on a per-trial basis. Therefore, both an objective intrinsic property of the auditory stimulus (i.e., the consonance), as well as a subjective measure of pleasure of the joint outcome significantly influences how participants synchronize their movements to each other, affecting the temporal coordination of their actions. Interestingly, the effect of consonance was stronger for dyads that reported feeling less close at the beginning of the experiment. Finally, we corroborate previous findings, by demonstrating a significant effect of musical training (even in non-musician participants) on interpersonal synchronization (Pecenka and Keller, 2011), thus supporting the validity of our measure in accurately assessing tapping production abilities in an interpersonal context. Together these findings suggest that the pleasantness of the joint auditory outcomes positively influences the accuracy of interpersonal synchronization, highlighting the importance of perceptual and aesthetic emotional factors in collaborative motor tasks.

Our findings indicate a significant relationship between the acoustic properties of the joint outcome and interpersonal synchronization (aim 1). The observed greater dyadic synchronization accuracy for consonant chords compared to dissonant ones suggests that the sensory-driven quality of what we produce together directly influences interpersonal motor coordination. Indeed, in our case, predictions about the quality of the joint outcome are purely driven by incoming perceptual information, since participants were unaware of the chords in advance, ruling out top-down expectations or strategic influences on their behavior. These results can be interpreted in light of the processing advantages for consonance compared to dissonance (Bones et al., 2014; Tabas et al., 2019; for a review, see Di Stefano et al., 2022). At the neural level, data has shown that consonant vs. dissonant stimuli are processed differently at both subcortical (Fishman et al., 2001; McKinney et al., 2001; Tramo et al., 2001; Bidelman and Krishnan, 2009) and cortical levels (Itoh et al., 2003, 2010; Bidelman and Grall, 2014) of the auditory system. These neurobiological studies have demonstrated that consonance processing begins early in the human auditory cortex and that additional neural resources are recruited to encode and discriminate dissonant chords compared to consonant ones (Tervaniemi et al., 2011; Virtala et al., 2013; Crespo-Bojorque et al., 2018). Interestingly, this distinctive activation pattern is observed in both humans and monkeys, suggesting a shared evolutionary trait (Fishman et al., 2001; Kadia and Wang, 2003; Bendor and Wang, 2005). The advantages of consonance extend beyond perceptual processing to impact higher-level cognitive abilities and motor performance. Crespo-Bojorque and Toro (2016) found that learning of stimulus-response association rules is facilitated when conveyed through consonant rather than dissonant intervals, while Komeilipoor et al. (2015) demonstrated that individual movement performance is less variable and more precise following exposure to a consonant as compared to a dissonant metronome. Our results align with these findings, showing that creating consonance together affects how we motorically synchronize with partners, thereby extending previous research to highlight the social impact of consonance. Minati et al. (2009) also observed strong right hemisphere activation (including premotor cortex and inferior parietal lobe) in response to consonant sounds. These brain regions are part of the dorsal auditory stream, which integrates auditory and motor information (Rauschecker, 2011; Lega et al., 2016). This neural pathway is particularly active in the right hemisphere for both rhythm perception (Chen et al., 2008; Siman-Tov et al., 2022) and production (Giovannelli et al., 2014). Moreover, other brain areas activated by consonant sounds, such as the orbitofrontal cortex, amygdala, and anterior cingulate gyrus (Dellacherie et al., 2009; Omigie et al., 2015), coincide with regions engaged in social behavior during interpersonal task (Beer et al., 2006; Cacioppo et al., 2014, see also aim 4). Taken together, this neural overlap between areas involved in consonance processing and interpersonal interaction bolsters the picture emerging from our study that these processes are linked.

Building upon the established link between consonance and enhanced synchronization, our findings underscore the significance of subjective pleasure in shaping interpersonal coordination. The observation that dyads' self-reported pleasure rating of the joint outcome predicts synchronization accuracy on a trial-by-trial basis highlights the interplay between perception and aesthetic pleasure in motor control. Previous studies have shown that negative interpersonal perception disrupts mutual motor adjustments (Sacheli et al., 2012) while improving synchronization (lower movement correction and variability). This suggests that partners who report a negative interpersonal bond execute a cooperative task more individually, less adapting to each other's motor behavior. Similarly, recent studies have experimentally manipulated emotional states (positive, negative, or neutral) and demonstrated that individuals induced with positive emotions, as opposed to negative emotions or a neutral state, maintained behavioral synchrony with other group members for a longer period of time (Smykovskyi et al., 2022). In contrast, inducing negative emotions significantly reduced the time spent in synchrony and decreased levels of synchronization (Smykovskyi et al., 2024). We speculate a similar mechanism may be at work in the present study, where the positive affective experience plausibly generated by consonant chords may promote more precise interpersonal movement coordination (i.e., mutual adaptation), but not necessarily improve the precision of the performance itself (i.e., individual synchronization with the metronome). Following this reasoning, we might hypothesize that consonance acts as a mediator of a pleasant affective experience, which in turn affects interpersonal motor coordination. Indeed, we showed that consonant chords received higher ratings of pleasure compared to dissonant ones, in line with a host of prior studies (Koelsch et al., 2006; Sammler et al., 2007; Krumhansl and Cuddy, 2010; McDermott et al., 2010; Komeilipoor et al., 2015). However, it is important to note that the present study design does not allow us to definitively disentangle the specific contributions of low-level perceptual features (consonance) and higher-level aesthetic experiences to the observed effects. Future studies employing more complex musical stimuli are necessary to test the selective contribution of these factors and to further explore the causal relationship between pleasure and interpersonal synchronization. Indeed, although we confirm that consonant chords were rated higher than dissonant ones, the levels of pleasure experienced by the presentation of single chords composed by pure tones are limited, as demonstrated also by the low variability of chord ratings of pleasure. Future studies could investigate full-fledged musical stimuli that presumably evoke more intense experiences of pleasure (see Blood and Zatorre, 2001; Salimpoor et al., 2011).

Interestingly, consonance affects synchronization between individuals but not individual tapping metrics, suggesting that the effect of consonance is primarily social in nature (aim 2). This result may seem to contradict the study by Komeilipoor et al. (2015), which demonstrated that individual motor synchronization performance, when the metronome was discontinued, was less precise and showed greater variability in the dissonant (vs. consonant) condition. In our study, consonance effects during individual tapping in the continuation phase did not reach significance, and, despite the significant interaction between consonance and auditory feedback duration, no consonant vs. dissonant differences were found in any feedback duration conditions. Thus, consonance did not affect overall individual synchronization with the metronome. Indeed, in our study participants were explicitly instructed to synchronize with each other, emphasizing the interpersonal aspect over individual synchronization, which may lead to the different outcomes compared to Komeilipoor et al. (2015), where participants tested alone were instructed to synchronize to a metronome. Previous studies have shown that when people engage in joint actions, top-down rule-based mechanisms can regulate bottom-up sensory-driven processes (Konvalinka et al., 2010). Specifically, when participants are instructed to perform a joint action, they mutually and continuously adapt their tap intervals, employing a “mutual adaptation” strategy (Konvalinka et al., 2010; Nowicki et al., 2013; Van Der Steen and Keller, 2013; Keller et al., 2014; Uccelli et al., 2023). Furthermore, in the study by Komeilipoor et al. (2015), consonance was not generated by the participants' movements but was instead delivered by an external stimulus beyond their control, a crucial difference that may help explain the divergent outcomes between their study and ours.

When investigating if reward sensitivity affects consonance, we anticipated that individuals more sensitive to aesthetic outcomes would show a more pronounced difference between consonant and dissonant sounds (aim 3). Although we did not find a significant correlation, the direction of the effect followed our expectations. Future research could delve further into this relationship, particularly examining which stages of the interaction between joint outcome and interpersonal synchronicity are most influenced by reward sensitivity (e.g., consonance, pleasantness, or beauty in general).

Our results indicate that the effect of consonance has a social component, as it is significantly modulated by the quality of the dyadic relationship prior to the experiment (aim 4). The direction of this relation was opposite to what we had hypothesized. Specifically, we demonstrated that the impact of consonance on interpersonal synchronization is greater in dyads that reported feeling less close before the task. We do not have a definitive explanation for this finding, and given that it was opposite to our hypothesis, we think further confirmatory experiments are needed to decide if this effect is robust. However, we might speculate on a potential underlying mechanism: individuals who already feel closer rely less on their joint outcomes to guide their behaviors, because the prior closeness buffers the need for a pleasurable outcome. By analogy, close friends may feel more at ease to have tough (not pleasurable) conversations because of the strength of their social bond. A limitation of this explanation is that the participants in our study were recruited specifically to not know each other beforehand, and hence the level of closeness would be limited. Individuals with less close interpersonal relationships may benefit more from positive external stimuli, such as consonant and pleasant interactions, to improve their emotional state and sense of connectedness (Lee et al., 2013; Taruffi and Koelsch, 2014; Schäfer et al., 2020). While the bidirectional relationship between perceived closeness and interpersonal synchronization has been previously established (Hove and Risen, 2009; Basile et al., 2022; Hu et al., 2022; Bégel et al., 2024), our results raise a possibility that this relationship could be mediated by the aesthetic experience of what is created together. Future studies should further explore these interactions and their causal direction, such as by manipulating the quality of the dyadic relationship and examining the effect of consonance on dyadic synchronization tasks.

## 5 Conclusion

In conclusion, our study demonstrates that the aesthetic quality of collaboratively produced sounds significantly influences the precision of interpersonal motor synchronization. These findings build on previous research examining factors such as tempo, timbre, and intensity in rhythmic joint actions, and highlight the importance of considering aesthetic and consonant elements in collaborative motor tasks. From a clinical perspective, these results are particularly valuable. If consonant musical pitch intervals can enhance movement synchronization more effectively than dissonant intervals, future research could leverage these stimuli for treating neurological and psychiatric disorders. By promoting the joint creation of pleasant sounds and synchronized movements, these techniques could improve movement performance in patients with sensory-motor deficits, such as Parkinson's disease (Rodger et al., 2014; Komeilipoor et al., 2015). Additionally, considering that schizophrenic patients often exhibit reduced synchronous behaviors, impaired movement and gestures, and social-affective disorders, pitch-based interpersonal synchronization tasks could help improve movement synchronization, foster feelings of closeness, and enhance social interactions (Varlet et al., 2012; Lavelle et al., 2014; Raffard et al., 2015; Dean et al., 2021).

## Data Availability

The datasets presented in this study can be found in online repositories. The names of the repository/repositories and accession number(s) can be found at: https://osf.io/y2aeh/?view_only=9e5991a23b414881a39cc678e7678ba9.
