# Supplementary material for: Pleasantness makes a good time: musical consonance shapes interpersonal synchronization in dyadic joint action
Source: Front Hum Neurosci. 2024 Oct 22;18:1472632. doi: 10.3389/fnhum.2024.1472632 (PMC11534602; doi:10.3389/fnhum.2024.1472632)
Supplement: Supplementary file 1 [file Data_Sheet_1.PDF]

Supplementary Material

S1. Subjective ratings of pleasure

Table S1. Effects of consonance, metronome IOI and auditory feedback duration on subjective ratings of pleasure. Results are graphically illustrated in Figure 2.

	**F**	**df**	**p**	**η^2_G**
Consonance	7.61	1,41	.009*	.02
Metronome IOI	1.30	2,82	.278	.004
Auditory feedback duration	15.39	2,82	<.001*	.11
Consonance x Metronome IOI	1.32	2,82	.272	.002
Consonance x Auditory feedback duration	6.15	2,82	.003*	.01
Metronome IOI x Auditory feedback duration	14.74	4,164	<.001*	.05
Consonance x Metronome IOI x Auditory feedback duration	2.77	4,164	.029*	.01

S2. Interpersonal synchronization (dSCT)

Table S2. Effects of consonance, metronome IOI, auditory feedback duration and task phase on the interpersonal synchronization (measured as mean of absolute taps difference in log ms) in the dSCT. Results are graphically illustrated in Figure S2.

	**F**	**df**	**p**	**η^2_G**
Consonance	7.99	1,20	.010*	.01
Metronome IOI	6.35	2,40	.004*	.05
Auditory feedback duration	8.16	2,40	.001*	.07
Task Phase	1.99	1,20	.173	.01
Consonance x Metronome IOI	2.37	2,40	.107	.003
Consonance x Auditory feedback duration	.83	2,40	.443	.002
Metronome IOI x Auditory feedback duration	.16	4,80	.960	.001
Consonance x Task phase	1.09	1,20	.308	.001

Metronome IOI x Task phase	1.87	2,40	.168	.01
Auditory feedback duration x Task phase	6.74	2,40	.003*	.01
Consonance x Metronome IOI x Auditory feedback duration	1.56	4,80	.193	.01
Consonance x Metronome IOI x Task phase	1.09	2,40	.347	.001
Consonance x Auditory feedback duration x Task phase	.97	2,40	.387	.001
Metronome IOI x Auditory feedback duration x Task phase	2.69	4,80	.037*	.01
Consonance x Metronome IOI x Auditory feedback duration x Task phase	1.11	4,80	.359	.003

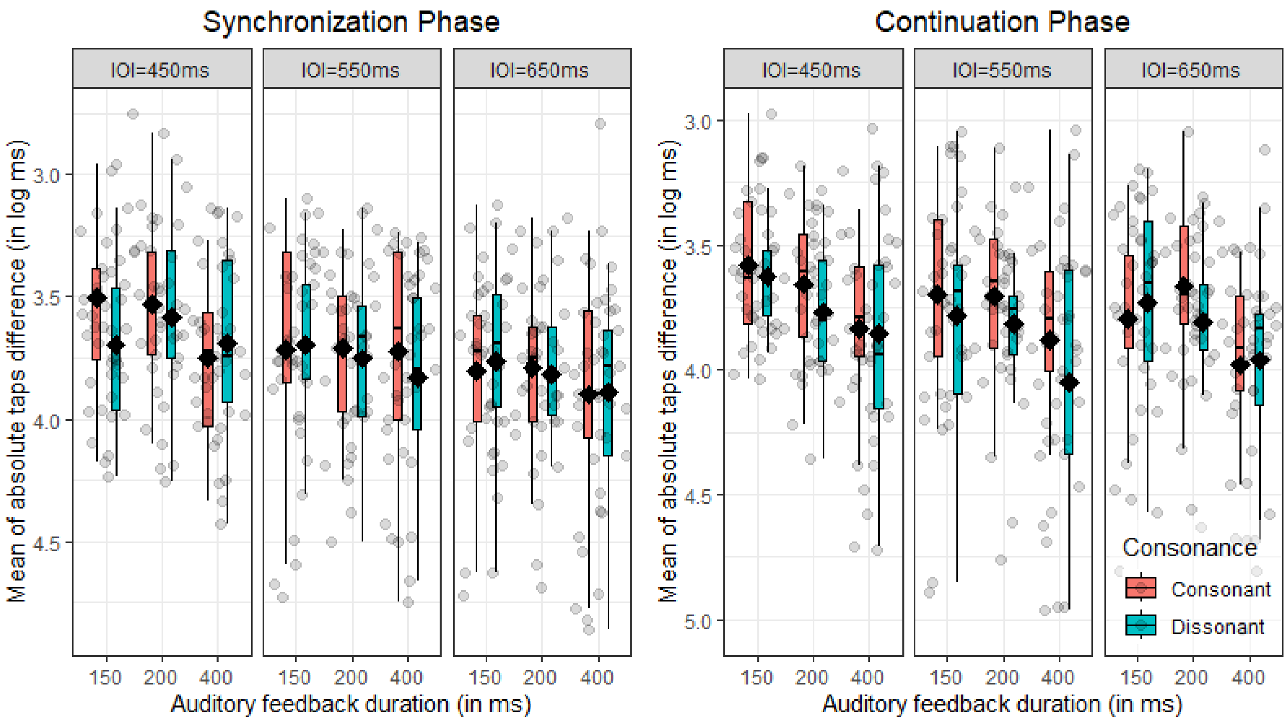

Figure S2. Plot of auditory feedback duration (x-axis; 150 ms, 200 ms and 400 ms), metronome Inter-Onset Intervals (split; 450 ms, 550 ms, or 650 ms), consonance (color; consonant vs. dissonant), divided in task phases (synchronization vs. continuation) on the interpersonal synchronization (measured as the mean of absolute difference between taps log transformed). Diamonds indicate the mean for each condition, while dots refer to the dyadic mean of absolute taps difference for each combination of conditions. Higher points suggest better interpersonal synchrony.

S3. Individual timing precision (dSCT)

A. Synchronization Phase

Table S3A. Effects of consonance, metronome IOI, auditory feedback duration on the individual tap timing relative to metronome (mean and variability) in the synchronization phase. Results are graphically illustrated in Figure S3A.

	**Model**	**F**	**df**	**p**	**η^2_G**
Consonance	*Means*	2.42	1,41	.127	.003
	SDs	3.32	1,41	.076	.003
Metronome IOI	*Means*	84.36	2,82	< .001*	.36
	SDs	39.90	2,82	< .001*	.27
Auditory feedback duration	*Means*	9.17	2,82	< .001*	.04
	SDs	12.95	2,82	< .001*	.06
Consonance x Metronome IOI	*Means*	.05	2,82	.952	< .001
	SDs	1.21	2,82	.305	.001
Consonance x Auditory feedback duration	*Means*	1.23	2,82	.296	.002
	SDs	.71	2,82	.495	.001
Metronome IOI x Auditory feedback duration	*Means*	.64	4,164	.636	.003
	SDs	3.01	4,164	.019*	.01
Consonance x Metronome IOI x Auditory feedback duration	*Means*	1.65	4,164	.164	.01
	SDs	1.61	4,164	.174	.01

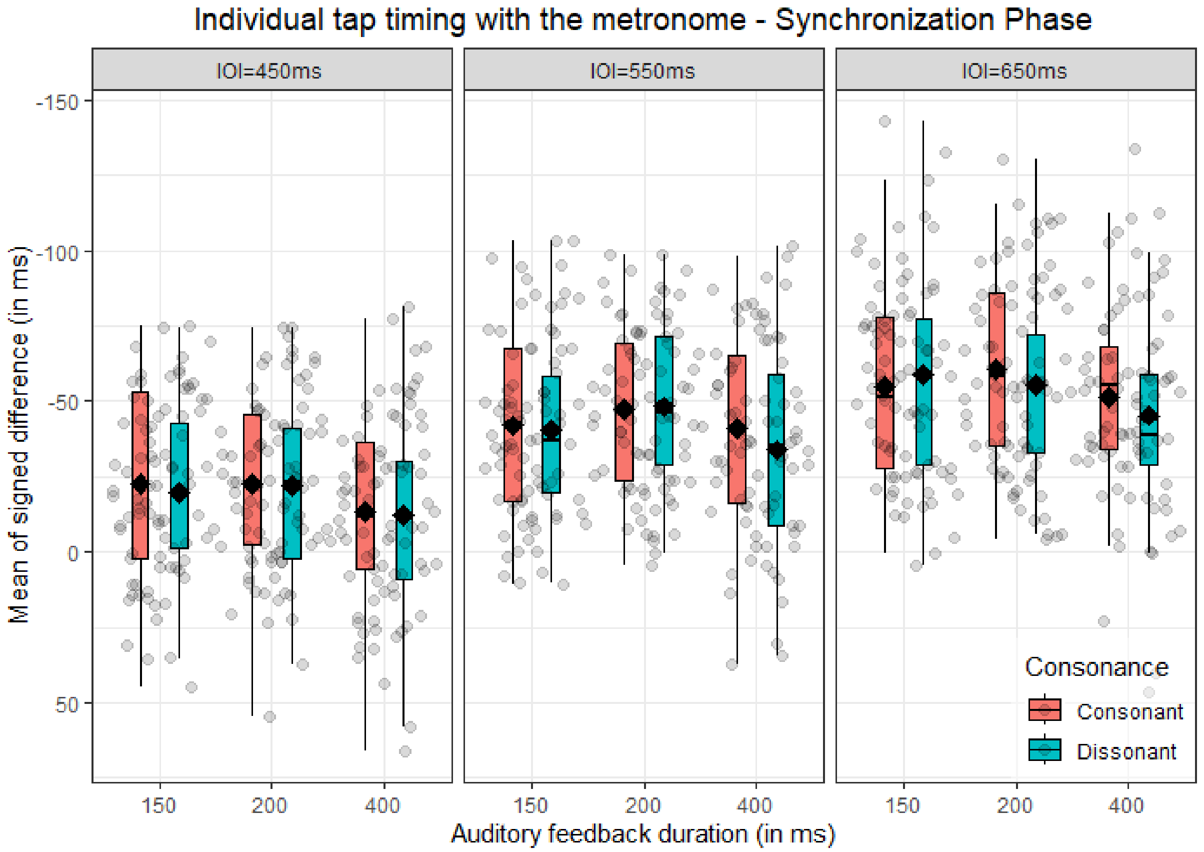

Figure S3A. Boxplot of auditory feedback duration (x-axis; 150 ms, 200 ms and 400 ms), consonance (color; consonant vs. dissonant) split by metronome Inter-Onset Intervals (450 ms, 550 ms, or 650 ms) on the individual timing relative to the metronome (measured as the mean of signed difference in ms) in the synchronization phase. Diamonds indicate the mean for each condition, while dots refer to the individual mean of signed difference for each combination of conditions. Values = 0 reflect perfect synchronization with the metronome.

B. Continuation Phase

Table S3B. Effects consonance, metronome IOI, auditory feedback duration on the individual timing (mean and SD Inter-Tap Intervals) in the continuation phase. Results are graphically illustrated in Figure S3B.

	Model	F	df	p	η^2_G
Consonance	*Means*	.07	1,41	.789	< .001
	SDs	3.81	1,41	.057	.003
Metronome IOI	*Means*	2760.17	2,82	< .001*	.96
	SDs	62.65	2,82	< .001*	.19

Auditory feedback duration	*Means*	86.77	2,82	< .001*	.27
	SDs	.19	2,82	.822	.001
Consonance x Metronome IOI	*Means*	1.34	2,82	.268	.001
	SDs	1.13	2,82	.327	.004
Consonance x Auditory feedback duration	*Means*	3.83	2,82	.026*	.01
	SDs	1.36	2,82	.262	.003
Metronome IOI x Auditory feedback duration	*Means*	20.93	4,164	< .001*	.07
	SDs	1.36	4,164	.250	.01
Consonance x Metronome IOI x Auditory feedback duration	*Means*	1.62	4,164	.173	.01
	SDs	1.47	4,164	.213	.01

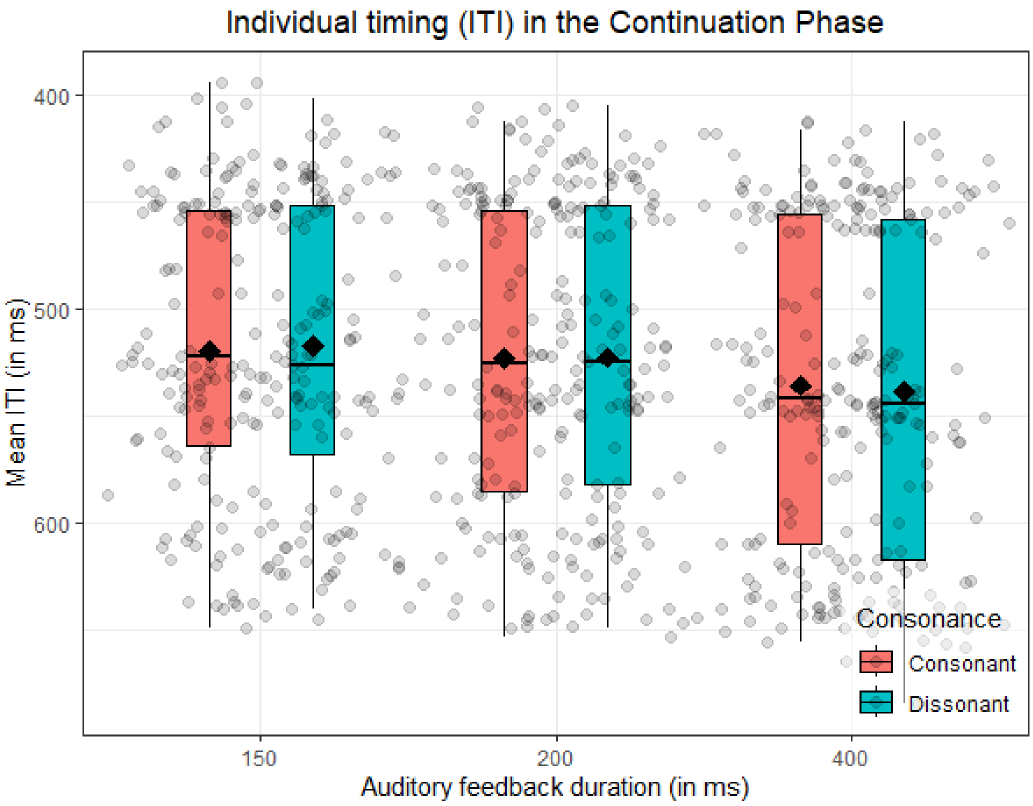

Figure S3B. Boxplot of the mean Inter-Tap Intervals (ITI) as a function of consonance (consonant vs. dissonant) and auditory feedback duration (150 ms, 200 ms and 400 ms). Diamonds indicate the mean ITI for each condition, while dots refer to the single participants' mean ITI for that specific combination of conditions.

S4. Analysis on the individual spontaneous tapping rate.

For the spontaneous tapping task, we analyzed mean ITI to measure the preferential tapping rate and to see how much participant-A's spontaneous pace differed from participant-B's. Thus, we computed a regression model predicting interpersonal synchronization (measured as the mean of absolute taps difference in log ms) by the delta of mean ITI. Our analysis revealed that spontaneous dyadic tapping rate does not significantly predict interpersonal synchronization (b = <.001, t = -.75, p = .462, R^2 = .03). Tranchant and colleagues (2022) and Zamm and colleagues (2016) found that individuals in dyads with similar spontaneous tapping rates exhibit enhanced synchrony in joint production tasks. However, our findings did not support this hypothesis. Unlike these previous studies, we specifically recruited only non-musicians, which could have contributed to the disparity in results. Additionally, participants performed a spontaneous tapping task without auditory feedback, whereas the effect observed in those studies was only significant when a melody was used as auditory feedback (e.g., "Twinkle"), which differed from the setup in our experiment.

S5. Analysis on the interpersonal tapping stability in the continuation phase.

We investigated interpersonal synchronization in the continuation phase of the dSCT. To account for drifts in tapping speed, we fitted a regression line to the Inter-Tap Intervals (ITIs) over time and reported the slope of this line as an indicator of tempo drift (van Vugt & Tillman, 2014). We calculated the intervals between consecutive taps of the same participant (ITIs), with ITIs reduction over time referring to anticipation of the tapping. Thus, we computed a within-dyads ANOVA with ITIs as the dependent variable and with consonance (2 levels: consonant vs. dissonant) and time (continuous variable from 1 to 20 taps in the continuation phase) as factors. If the mean of absolute taps difference provides a measure of precision in synchronizing one another, the slope analysis is useful to specifically supply a temporal index of consistency and stability related to the imaged metronome in the continuation phase. Results showed neither a main significant effect of consonance (F(1,20) = .11, p = .749, η^2_G = .001) nor an interaction consonance by time (F(19,380) = .74, p = .778, η^2_G = .01). The main effect of time turned out statistically significant (F(19,380) = 13.25, p <.001, η^2_G = .26), indicating that participants tended to anticipate the extrapolated timing of the metronome more as time passes (from M_{first_ITI} = 537.48 ms, SD = 80.91 ms, to M_{last_ITI} = 519.67 ms, SD = 82.10 ms), as expected. As time progressed, participants demonstrated a tendency to anticipate the metronome, consistent with findings from prior research indicating a more pronounced Negative Mean Asynchrony (NMA) in dyadic settings (Kovalinka et al., 2009; 2010; Mates et al., 1992; Nowicki et al., 2013).

S6. Assessment of psychological constructs

Table S6. Correlations (A) between consonance effect and questionnaire dyadic scoring sum and (B) between interpersonal synchronization and questionnaire dyadic sum (see Figure S6).

**A) Consonance effect**		**r**	**p**	**B) Interpersonal Synchronization**		**r**	**p**
	IOS pre	.46	.037*		IOS pre	-.04	.864
	IOS post	.17	.469		IOS post	-.24	.298
	post-pre IOS	-.39	.078		post-pre IOS	-.27	.242
	AQ	.01	.981		AQ	-.27	.232
	eBMRQ	-.33	.139		eBMRQ	-.19	.415
	Musical Training	-.09	.674		Musical Training	-.59	.005*
	Perceptual Abilities	-.09	.684		Perceptual Abilities	-.36	.105

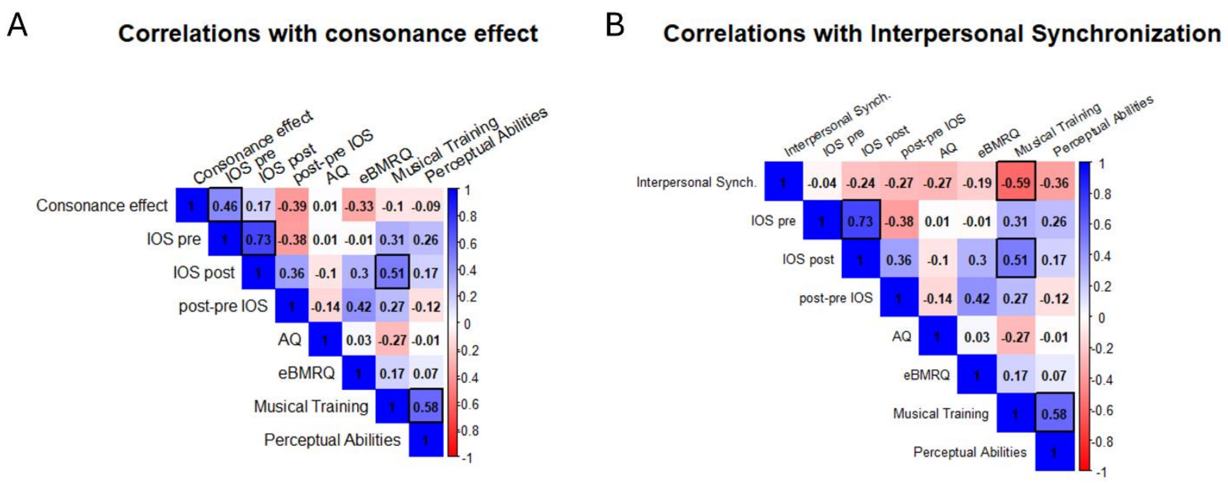

Figure S6. Correlation matrices (A) between consonance effect and questionnaire dyadic sum and (B) between interpersonal synchronization and questionnaire dyadic sum. Highlighted squares represent significant correlations (see Table S6).
